# The versatile thoracodorsal artery perforator flap for extremity reconstruction: from simple to five types of advanced applications and clinical outcomes

**DOI:** 10.1186/s13018-023-04480-3

**Published:** 2023-12-18

**Authors:** Xinlei Sui, Liming Qing, Fang Yu, Panfeng Wu, Juyu Tang

**Affiliations:** Department of Orthopaedics, Xiangya Hospital, Central South University, Changsha, 410008 China

**Keywords:** Thoracodorsal artery perforator flap, Microsurgery, Extremity reconstruction, Clinical outcomes

## Abstract

**Background:**

Application of the thoracodorsal artery perforator (TDAP) flap is known to be a popular and reliable method for extremity reconstruction. This manuscript presents our clinical outcomes in reconstructing soft tissue defects using simple and advanced TDAP flaps.

**Methods:**

From 2013 to 2022, 53 patients with a mean age of 23 years (ranging from 2 to 72 years) underwent reconstructive surgery with different patterns of free TDAP flaps, including chimeric TDAP flaps, double skin paddle TDAP flaps, flow-through TDAP flaps, conjoined TDAP flaps, and microdissected debulking TDAP flaps.

**Results:**

All TDAP flaps survived. The size of the TDAP skin paddle ranged between 5 × 3 and 25 × 10 cm^2^. Primary closure of the donor site was achieved in all patients in the simple application group, and one patient in the advanced application group underwent partial skin grafting. Partial flap loss occurred in one case in the simple TDAP flap group and four cases in the advanced application group. There was one case of flap bulkiness and two cases of scar hyperplasia in the simple TDAP flap group. The mean follow-up duration was 11 months (4–46 months).

**Conclusions:**

The free TDAP flap, with five types of advanced applications, makes it versatile for reconstructing different kinds of soft tissue defects of the extremities that can be used to achieve individualized defect reconstruction, minimize donor site morbidities, and an aesthetic appearance.

**Supplementary Information:**

The online version contains supplementary material available at 10.1186/s13018-023-04480-3.

## Introduction

Complex defects of the extremities frequently arise in clinical settings and are generally caused by trauma, lesion resection, and congenital abnormalities [1–4]. With the development of the perforator flap, reconstructive surgeons pursue not only flap survival but also improved appearance of the recipient site and reduced damage to the donor site [5–7]. Regarding different wounds, featureless flap transplantation is no longer performed. More appropriate donor sites are individually selected and the flap design is optimized for wound repair according to the defect characteristics.

The anterolateral thigh (ALT) flap has always been a classic flap in soft tissue reconstruction, which can provide a large area of skin and muscle with constant anatomy and reliable blood supply [8–10]. However, when the patient is overweight, the harvested skin flap is usually thick and unsuitable for covering superficial wounds on the limbs. Additionally, scarring on the thigh is not aesthetically pleasing, especially for women. Therefore, the thoracodorsal artery perforator (TDAP) flap is a suitable alternative as it comes from a more hidden area.

The TDAP flap is also commonly used to repair soft tissue defects of the extremities and was first reported by Angrigiani in 1995 [11]. It was soon widely applied because of its advantages, such as the hidden donor site, convenient flap harvesting, and constant anatomy [12–15]. In this paper, we reported our clinical outcomes of reconstructing different wounds of the extremities with different applications of TDAP flaps from simple to five types of advanced patterns.

## Patients and methods

### Patients

This retrospective study enrolled 53 patients aged between 2 and 72 years (25 males and 28 females) who underwent reconstructive surgery with different types of TDAP flaps from January 2013 to December 2022. The cause of injury or etiology included traffic accident (*n* = 24), machine injury (*n* = 12), scar release (*n* = 7), chronic ulcer (*n* = 5), tumor resection (*n* = 4), and explosive injury (*n* = 1). The defect sites were located on the limbs and trunk. The detailed patient characteristics are listed in Table 1.

**Table 1:** Patient characteristics

Variable	Value (%)
No. of patients	53
Age (year)	
Mean	23
Range	2–72
Sex	
Male	25 (47)
Female	28 (53)
Etiology	
Traffic accident	24 (45)
Machine injury	12 (23)
Scar release	7 (13)
Chronic ulcer	5 (9)
Tumor resection	4 (8)
Explosive injury	1 (2)
Defect location	
Upper arm and forearm	5 (9)
Hand and wrist	11 (21)
Thigh and lower leg	20 (38)
Foot and ankle	17 (32)
Type of TDAP flap	
Simple application	17 (32)
Advanced application^a^	36 (68)
Chimeric	30 (70)
Microdissected debulking	6 (14)
Flow-through	4 (9)
Double skin paddle	2 (5)
Conjoined	1 (2)
Follow-up month (month)	
Mean	11
Range	4–46

*TDAP* thoracodorsal artery perforator

^a^Some patients received more than one advanced application

The same surgical team performed all operations. All patients and their families provided written informed consent. Ethics approval was obtained from the local ethics committee.

### Surgical methods

Preoperatively, computed tomography angiography (CTA) was conducted in all patients to map the perforators. A handheld Doppler probe was applied to locate the perforators.

The TDAP flap was harvested as described in our previous study [16, 17]. Briefly, a paper template was tailored according to the shape of the skin defects after radical debridement. The design of the flap was outlined on the thoracodorsal site based on the perforators detected preoperatively. The flap was then elevated from the lateral border. The main perforator was defined during dissection above the deep fascia. Then, the perforator was retraced back to the main trunk of the thoracodorsal artery (TDA) and dissected at the proper position according to the needed pedicle length.

**Fig. 1:**
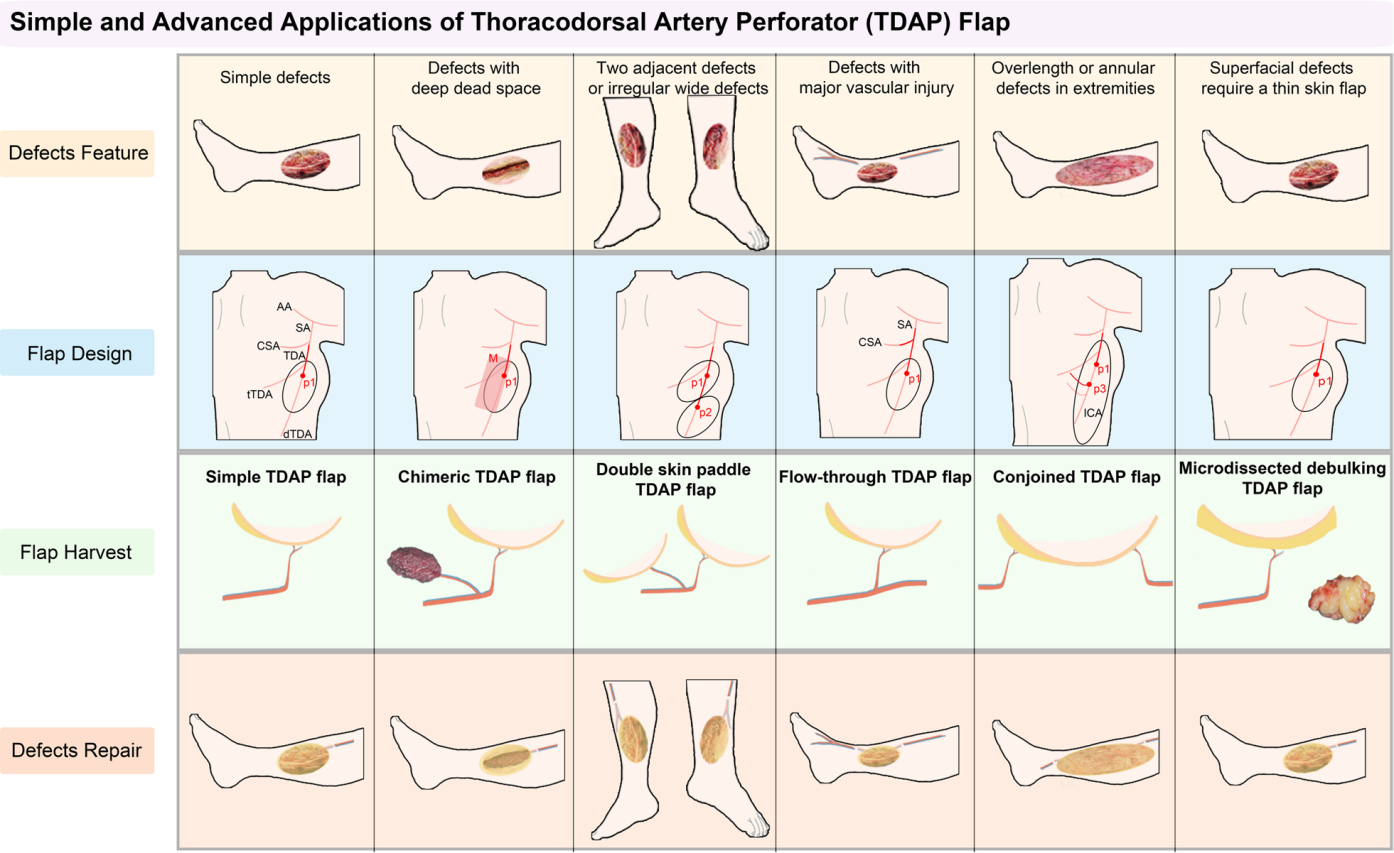
Simple and advanced applications of TDAP flap. The simple and five advanced applications of TDAP flap can be harvested according to the defect features. *TDAP* thoracodorsal artery perforator, *AA* axillary artery, *SA* subscapular artery, *CSA* circumflex scapular artery, *TDA* thoracodorsal artery, *tTDA* transverse branch of TDA, *dTDA* descending branch of TDA, *ICA* intercostal artery, *p1* primary perforator of the thoracodorsal artery, *p2* secondary perforator of the thoracodorsal artery, *p3* perforator of the intercostal artery, *M* latissimus dorsi muscle paddle

The following five advanced patterns of TDAP flaps can be harvested according to the defect characteristics (Fig. 1).

#### The chimeric TDAP flap

This flap consists of a TDAP skin paddle and a latissimus dorsi muscle paddle supplied by the independent branches of the TDA, and the flap can be revascularized by anastomosing a group of pedicles. It is suitable for repairing defects with deep dead space. During the dissection of the perforators, the thick muscle branch is retained, and the muscle paddle is harvested to an appropriate size. Then, the muscle branch is retraced to the main trunk of the TDA.

#### The double skin paddle TDAP flap

This flap consists of two separate skin paddles nourished by independent perforators of the TDA, and the blood supply can be restored by anastomosing a set of pedicles. Two main perforators in each paddle are dissected to the TDA trunk. For two adjacent defects, two skin paddles can cover the defects separately. For a wide defect, two paddles can be well-designed, harvested, and sutured together to conform to the shape of the defect.

#### The flow-through TDAP flap

The T-shaped pedicle is the main feature of this flap. Specifically, the subscapular artery and circumflex scapular artery are ligated and cut off at the proper position and separately anastomosed to the proximal and distal ends of the recipient vessels. This flap is suitable for wounds with primary segmental vascular defects.

#### The conjoined TDAP flap

This flap consists of an overlength TDAP flap harvested exceeding the angiosome of the TDA. To ensure flap survival, the perforator of the corresponding angiosome must be carried and reconstructed. The conjoined TDAP flap has two pedicles, a primary thoracodorsal vascular pedicle, and an accessory pedicle. In our case of the conjoined TDAP flap application, the accessory pedicle came from intercostal vessels.

#### The microdissected debulking TDAP flap

Removing excess subcutaneous adipose tissue from a thick flap allows more aesthetic reconstruction. The perforators in the superficial fascia are dissected and protected under a microscope or magnifying glasses, and approximately 5 mm of fascia around the perforators is preserved.

After harvesting a particular TDAP flap, the flap was transferred to the recipient site, and the thoracodorsal vessels were anastomosed to the proper recipient vessels. The donor site was then closed directly.

## Results

A total of 53 patients with a mean age of 23 years (range 2–72 years) underwent reconstruction with different TDAP flap patterns. Of the 53 patients, 17 (32%) were treated with a simple TDAP flap, and 36 (68%) were treated with an advanced TDAP flap. In some patients, more than one advanced pattern was applied. There was a total of 30 cases (70%) of chimeric flap use, six cases (14%) of microdissected debulking flap use, four cases (9%) of flow-through flap use, two cases (5%) of double skin paddle flap use, and one case (2%) of conjoined flap use (Table 1).

The size of the TDAP skin paddle ranged between 5 × 3 and 25 × 10 cm^2^. In the use of chimeric TDAP flaps, the size of the latissimus dorsi muscle paddle harvested was between 4 × 3 and 16 × 13 cm^2^. In all patients treated with simple TDAP flaps, primary closure of the donor site was achieved. Direct closure was not achieved in one patient treated with a chimeric and microdissected debulking TDAP flap, and this patient underwent skin grafting with a graft 5 × 3 cm^2^ in size (Table 2).

**Table 2:** Intraoperative and follow-up data

Variable	Simple application *n* = 17	Advanced application *n* = 36
Skin paddle size (cm^2^)	5 × 3 to 20 × 7	6 × 3 to 25 × 10
Muscle paddle size (cm^2^)	–	4 × 3 to 16 × 13
Donor site closure		
Primary closure	17	35
Partial skin grafting	0	1
Flap-associated complication		
Venous crisis	0	1
Partial flap loss	1	4
Total flap loss	0	0
Long-term complication		
Scar hyperplasia	2	0
Bulky flap	1	0
Secondary procedure		
Vascular exploration	0	1
Free flap transplantation	0	2
Skin grafting	1	0
Flap debulking	1	0

All the different patterns of TDAP flaps survived, but partial flap loss occurred in five cases (9%). One case of partial flap loss occurred in a simple TDAP flap due to the compression of a hematoma and was treated by skin grafting. Three cases occurred in the chimeric TDAP flaps; one was cured by routine dressing changes, and secondary flap transplantation was performed for the other two. Another case of partial flap loss occurred in a double skin paddle TDAP flap and was treated by dressing changes. In addition, a venous crisis occurred in one patient treated with a chimeric TDAP flap, and rescue was achieved by surgical exploration.

Long-term complications were observed in three cases (6%) of the simple TDAP flaps, including two cases of scar hyperplasia and one case of flap bulkiness. Secondary surgical flap debulking was performed 4 months after the reconstructive operation. Overall, the reoperation rate was 9%. The mean follow-up period was 11 months (4–46 months).

## Case reports

### Case 1

**Fig. 2:**
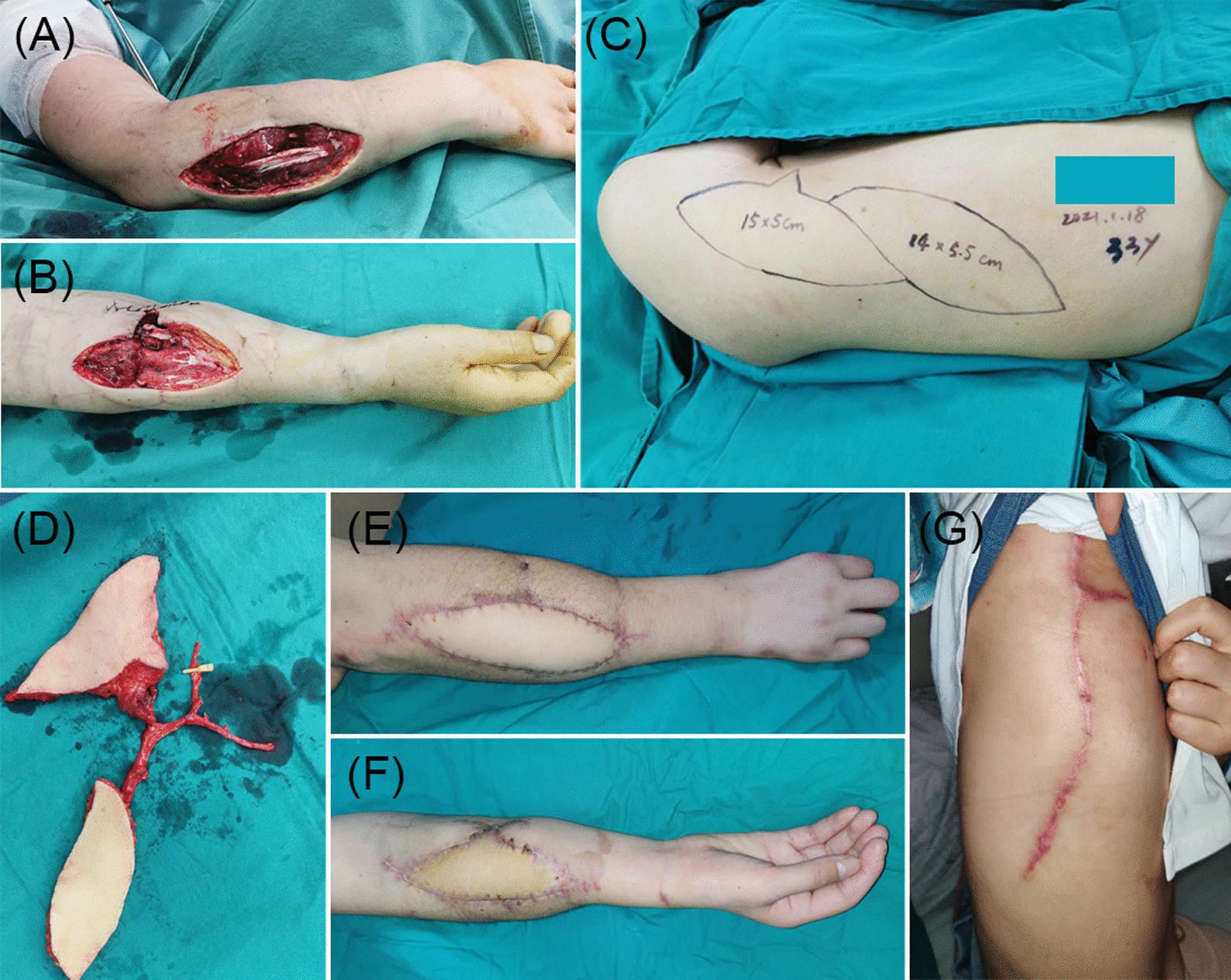
**A**, **B** A 33-year-old female presented with two adjacent skin and soft tissue defects on the medial and lateral sides of her right forearm. **C** Flap design of a double skin paddle TDAP flap. **D** A double skin paddle TDAP flap harvest. **E**–**G** Postoperative outcomes at 4 months after the reconstructive surgery

A 33-year-old female presented with two adjacent skin and soft tissue defects on the medial and lateral sides of her right forearm due to a machine injury (Fig. 2A, B). Reconstruction was performed using a double skin paddle TDAP flap (Fig. 2C, D), with anastomosis between the TDA and the radial artery. The donor site was closed directly. The flap survived successfully without complications. At 4 months postoperatively, both the donor and recipient sites presented a favorable appearance (Fig. 2E–G).

### Case 2

**Fig. 3:**
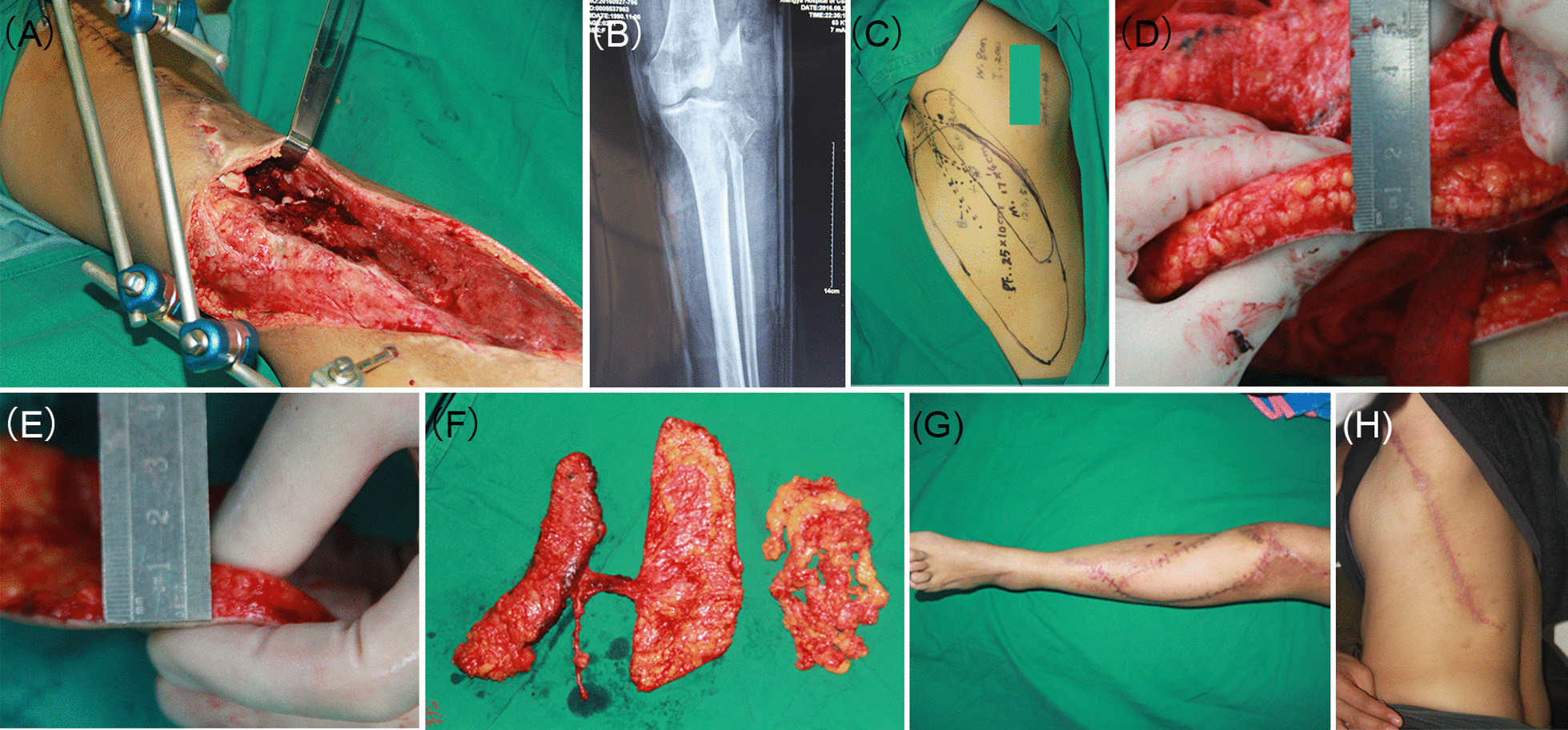
**A** Large skin defect with deep dead space after debridement and external fixation of the fractures. **B** Preoperative X-ray photograph. **C** Flap design of a chimeric and microdissected debulking TDAP flap. **D**, **E** Flap thickness before and after microdissected debulking. **F** The harvested chimeric and microdissected debulking TDAP flap. **G**, **H** Postoperative outcomes of the recipient and donor site at 5-month follow-up

A 25-year-old female suffered a traffic accident. After debridement and external fixation of the fractures, the patient presented skin defects of the knee and lower leg and deep tissue defects of the lateral structures of the knee, including the lateral condyle of the femur and tibia, iliotibial band, biceps femoris, and fibular collateral ligament. Partial defects of the above structures left a deep cavity (Fig. 3A, B). In addition, the patient had a thick layer of subcutaneous fat on her back. Therefore, a chimeric and microdissected debulking TDAP flap was designed according to the characteristics of the defects and the donor area (Fig. 3C). The thickness of the flap was reduced from 2 to 1 cm by debulking (Fig. 3D, E). Then, the flap was raised, and the vascular anastomosis was performed between the TDA and the medial sural artery (Fig. 3F). Primary closure of the donor site was achieved, and no complications occurred during the follow-up period. Five months after surgery, the recipient and donor areas presented a cosmetic appearance (Fig. 3G, H). Functionally, knee stability and walking performance exhibited significant recovery at 18 months postoperatively (Additional files 1 and 2: Videos 1 and 2).

### Case 3

**Fig. 4:**
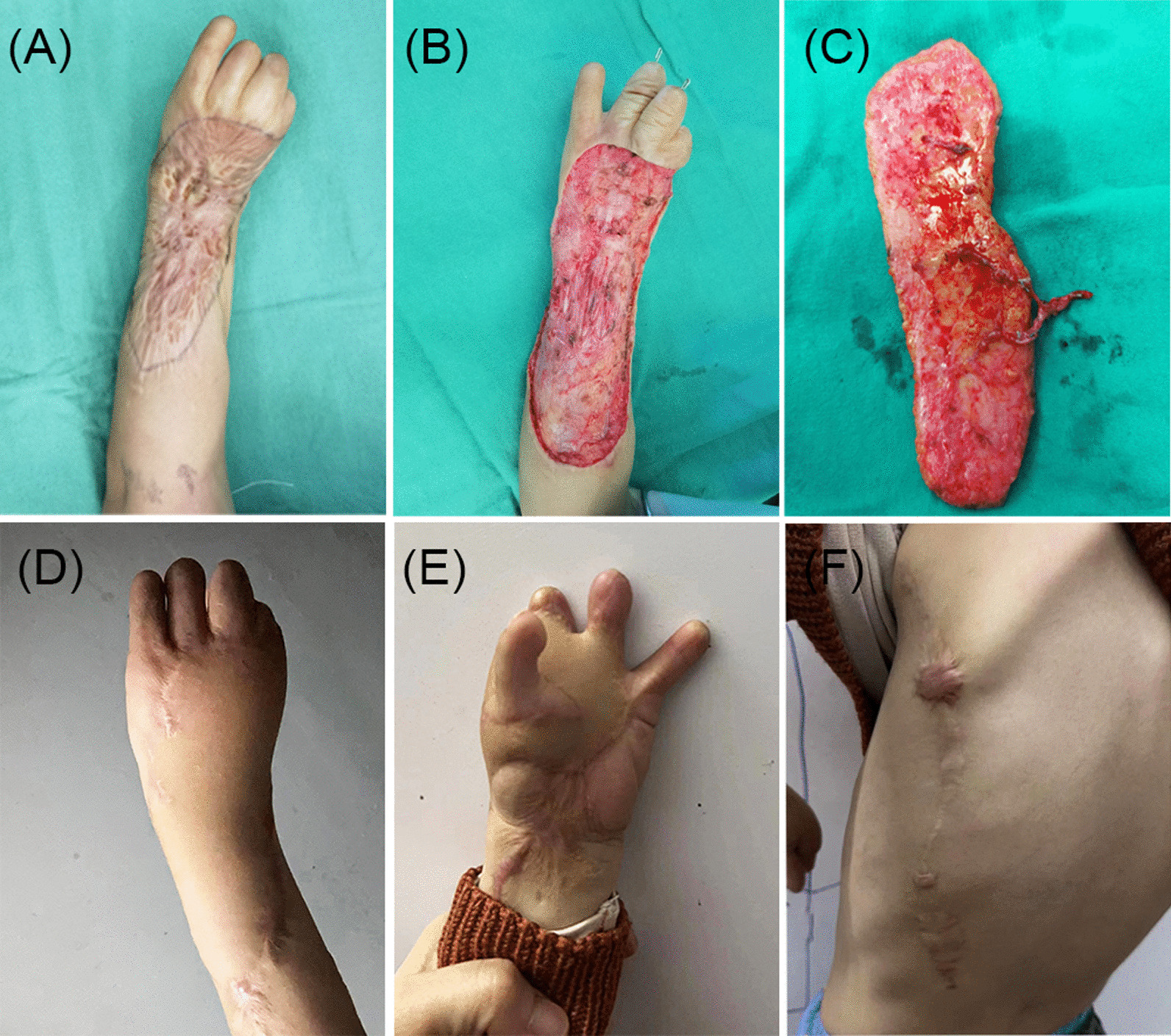
**A** A 3-year-old male patient suffered a scar contracture on his right hand. **B** An overlong skin and soft tissue defects after scar removal. **C** A flow-through conjoined TDAP flap harvest. **D**–**F** Postoperative outcomes of the recipient and donor site at 23-month follow-up

After scar removal and tendon release, a 3-year-old boy presented with an overlong skin and soft tissue defect on his right hand and forearm (Fig. 4A, B). A flow-through conjoined TDAP flap was applied (Fig. 4C). The subscapular artery was anastomosed to the proximal radial artery, and the circumflex scapular artery was anastomosed to the distal radial artery. In addition, the intercostal artery in the flap was anastomosed to a branch of the TDA as a supercharge. The donor site was closed directly, and the flap survived without complications. One year after the surgery, thumb reconstruction was conducted. At the final follow-up, the patient and his family were satisfied with the appearance and function of the affected areas (Fig. 4D–F).

## Discussion

Microsurgery has developed rapidly since Koshima first proposed the concept of the perforator flap in 1998 [18–21]. The goal of reconstructive surgeons has gradually upgraded from flap survival to aesthetic repair and minimal invasiveness. Different defects prompt continual consideration of better reconstructive strategies. When there are deep tissue defects, simple skin flap coverage will leave a dead cavity, resulting in hematoma and infection. When a wound is irregular and broad or there are two adjacent wounds, a single flap cannot meet the demand, while composite flap transplantation is costly and time-consuming. When the trauma is severe and there is a major segmental vascular defect, whether the vascular and soft tissue defects can be reconstructed by a simple method simultaneously without vessel grafting remains a question. When the patient is overweight, the flap is often bloated and seriously affects the appearance. These problems cannot be solved by simple perforator flap transplantation.

This paper reports our clinical experience in treating different wounds with multiple applications of TDAP flaps, from simple to five types of advanced patterns. To our knowledge, this is the most extensive series describing the various uses of TDAP flaps. Our results demonstrated the versatility of clinical applications of TDAP flaps with an individualized design and harvest strategy according to the different defect features.

The TDAP flap is currently a popular flap in the field of reconstruction because of its multiple advantages [22–24]. First, the anatomy of the TDA and its perforators are relatively constant and have a diameter similar to that of the main vessels of the limbs [25]. Second, both the skin and muscle paddle can be harvested in a large size. Furthermore, the donor site is hidden and can usually be closed directly, thus providing an aesthetic appearance and minimizing donor site morbidity [26]. In addition, the thoracodorsal nerve can be carried in the flap to restore the flap sensory [27]. However, a simple TDAP flap cannot solve the abovementioned problems. Thus, we summarized five types of advanced patterns of TDAP flaps to make the TDAP flap more useful in clinical practice.

The chimeric TDAP flap is created by the simultaneous harvest of a skin paddle and either a latissimus dorsi muscle paddle, a scapula bone paddle, or a rib bone paddle, all supplied by the TDA [28, 29]. In this study, a chimeric TDAP flap with a latissimus dorsi muscle paddle was the most commonly used advanced pattern among the 36 patients who underwent advanced TDAP flap transfer. It has obvious advantages in the treatment of wounds with deep tissue defects. Specifically, the skin paddle can cover superficial defects, and the muscle paddle can fill dead spaces. In this way, skin and deep tissue defects can be repaired simultaneously by anastomosing a group of blood vessels. In addition, the latissimus dorsi muscle has an abundant blood supply and a good capacity to resist infection [30]. Additionally, the muscle paddle can be accurately harvested according to the size of the dead space, which not only improves the quality of recipient site repair but also preserves the integrity of the latissimus dorsi to the greatest extent possible. Using a chimeric TDAP flap, the damage to the donor site is less than that of a traditional latissimus dorsi flap.

The double skin paddle TDAP flap consists of two distinct skin paddles, each nourished by independent perforators originating from the TDA and connected through the same TDA pedicle [31, 32]. This flap is suitable for reconstructing two adjacent wounds or wounds that are irregular and broad, which can be repaired by appropriate design, harvest, and recombination of the two paddles to conform to the shape of the defect. Meanwhile, primary closure of the donor site can be achieved. Notably, preoperative CTA or Doppler examination is necessary to map the perforators and avoid blind surgical procedures.

By applying a flow-through TDAP flap with a T-shaped pedicle, the subscapular artery is anastomosed to the proximal recipient artery, and the circumflex scapular artery is anastomosed to the distal recipient artery to reconstruct blood flow and avoid sacrificing the main blood vessels of the recipient site [33, 34]. This flap can be used for soft tissue defects with primary segmental vascular defects. In addition, the circumflex scapular artery can be anastomosed to a second flap to connect two flaps and achieve reconstruction of a larger area. Preoperative CTA examination is also helpful in this context to exclude vascular variation.

In the conjoined TDAP flap, the length of the skin flap exceeds the nourishing capacity of the angiosome of the TDA, necessitating an additional perforator from the corresponding angiosome to ensure flap survival [35, 36]. This flap is suitable for overlength or annular defects in the extremities. Notably, when only one group of vessels can be used for reconstruction at the recipient site, a flow-through conjoined TDAP flap whose proximal end should be anastomosed to the recipient vessels and distal end to the accessory pedicle should be harvested. If two groups of available recipient vessels exist, anastomosis with the primary and accessory pedicles can be performed separately.

For microdissected debulking TDAP flaps, the subcutaneous adipose tissues of the flap are removed while protecting the perforators in the superficial fascia and the dermal vascular network under the microscope [37]. This flap is suitable for overweight patients who suffer superficial skin and soft tissue defects of their limbs. In this way, an aesthetic and satisfied appearance can be achieved, and secondary debulking surgery can be avoided. However, dissecting the perforators within the superficial fascia is time-consuming and carries the risk of perforator injury.

In many situations, satisfactory reconstruction can be achieved by applying or combining five advanced forms of TDAP flaps after careful analysis of the defect features. For example, in case 2 in our study, we applied a chimeric and microdissected debulking TDAP flap. The muscle paddle filled the deep dead space, and the thinner skin paddle improved the appearance of the recipient area. Meanwhile, only one group of vessels needed to be anastomosed.

In our study, the overall complication rate was 17% (9/53). The rate of flap-associated complications was 11% (6/53), which could potentially be attributed to the severity of the defects, compromised vascular conditions, and the intricate nature of the surgical procedures. In the previous study, Guerra et al. [38] reported their experience using a free TDAP flap for soft tissue reconstruction, with a total complication rate of 29% (5/17) and a flap-associated complication rate of 12% (2/17, one case of flap loss and one case of partial loss). Kim et al. [39] reported 11 cases of free TDAP flap transplantation using their defatting technique with good results, in which two patients with a history of diabetes developed partial flap loss (2/11). In this study, the three cases of long-term complications occurred in the simple TDAP flaps, which may indicate the superiority of the advanced applications of TDAP flaps.

There are some limitations to the applications of TDAP flaps. First, the pedicle length is usually not long enough. Second, the TDA mostly has only one accompanying vein. When a large flap is transplanted, there is a risk of insufficient venous return, so an additional set of superficial veins needs to be anastomosed. In addition, the body position may need to be changed during the operation. Additionally, it is technically demanding to apply advanced TDAP flaps.

This study also has several limitations. The small number of patients treated with advanced patterns of TDAP flaps limits the ability to identify the associated complications and risk factors. Another limitation is the absence of a control set of patients. For instance, we did not compare the TDAP flap to other flaps to reconstruct the different defects. Additionally, there is a lack of standardized postoperative functional assessments. However, our growing experience with the five advanced patterns of the TDAP flap has led to broader indications for the TDAP flap in reconstructing soft tissue defects with different features.

## Conclusions

The free TDAP flap with five types of advanced applications is a versatile tool for reconstructing different kinds of defects in extremities. The chimeric TDAP flap is suitable for wounds with deep tissue defects. The double skin paddle TDAP flap is suited for repairing two adjacent or irregular, wide wounds. The flow-through TDAP flap can be used for injuries with major segmental vascular defects. The conjoined TDAP flap is suitable for overlength or annular wounds of the extremities. The microdissected debulking TDAP flap is appropriate to cover a superficial wound that requires a thin skin paddle. These procedures can be used to achieve individualized defect reconstruction while minimizing donor site morbidities and leading to an aesthetic appearance in both recipient and donor areas.

### Supplementary Information

**Additional file 1**. **Video 1**. Stability of lateral structures of the knee joint at 18 months after the reconstructive surgery.

**Additional file 2**. **Video 2**. Walking performance at 18 months after the reconstructive surgery.

## Data Availability

The data that support the findings of this study are available on request from the corresponding author.

## Abbreviations

ALT: Anterolateral thigh
CTA: Computed tomography angiography
TDA: Thoracodorsal artery
TDAP: Thoracodorsal artery perforator

## References

[1] Qing L, Wu P, Luo G (2022). Waveform-arranged multiple skin paddles: a novel design to reconstruct complex soft tissue defects of the extremities with a modified multi-lobed perforator flap. Injury.

[2] Ye SM, Yu Y, Jing JH (2019). One-stage reconstruction of complex soft tissue defects in the hands using multidigit, chimeric, lateral arm, perforator flaps. J Plast Reconstr Aesthet Surg.

[3] di Summa PG, Sapino G, Cherubino M (2019). Reconstruction of complex soft tissue defects including tendons with anterolateral thigh flap extended to fascia lata: long term recovery and functional outcomes. Microsurgery.

[4] Sahasrabudhe P, Panse N, Baheti B (2015). Reconstruction of complex soft-tissue defects around the knee joint with distally based split vastus lateralis musculocutaneous flap: a new technique. J Plast Reconstr Aesthet Surg.

[5] Opsomer D, Vyncke T, Ryx M (2022). Donor site morbidity after lumbar artery perforator flap breast reconstruction. J Reconstr Microsurg.

[6] Abdelfattah U, Power HA, Song S (2019). Algorithm for free perforator flap selection in lower extremity reconstruction based on 563 cases. Plast Reconstr Surg.

[7] Otani N, Tashima H, Tomita K, Kurita T, Kubo T (2020). Latissimus dorsi-lumbar artery perforator chimeric flap with intra-flap crossover anastomosis for breast reconstruction. Plast Reconstr Surg Glob Open.

[8] He J, Qing L, Wu P (2021). Customized reconstruction of complex soft tissue defects in the upper extremities with variants of double skin paddle anterolateral thigh perforator flap. Injury.

[9] Hsieh F, Leow OQY, Cheong CF, Hung SY, Tsao CK (2021). Musculoseptocutaneous perforator of anterolateral thigh flap: a clinical study. Plast Reconstr Surg.

[10] Zheng X, Zheng C, Wang B (2016). Reconstruction of complex soft-tissue defects in the extremities with chimeric anterolateral thigh perforator flap. Int J Surg.

[11] Suba S, Giri SK, Pant P, Kanungo A (2022). Versatility of medial sural artery islanded pedicled perforator flap for resurfacing areas around the knee. Ann Plast Surg.

[12] Brambilla L, Parisi P, Gatto A (2022). A retrospective comparative analysis of latissimus dorsi (LD) flap versus thoracodorsal artery perforator (TDAP) flap in total breast reconstruction with implants: a pilot study. J Reconstr Microsurg.

[13] Sung IH, Jang DW, Kim SW, Kim YH, Kim SW (2018). Reconstruction of diabetic lower leg and foot soft tissue defects using thoracodorsal artery perforator chimeric flaps. Microsurgery.

[14] Berna P, Toublanc B, Fourdrain A (2018). Successful bronchial replacement using a thoracodorsal artery perforator flap. Ann Thorac Surg.

[15] Ayhan S, Tuncer S, Demir Y, Kandal S (2008). Thoracodorsal artery perforator flap: a versatile alternative for various soft tissue defects. J Reconstr Microsurg.

[16] He J, Guliyeva G, Wu P (2022). Reconstruction of complex soft tissue defects of the heel with versatile double skin paddle anterolateral thigh perforator flaps: an innovative way to restore heel shape. Front Surg.

[17] Qing L, Wu P, Yu F, Zhou Z, Tang J (2020). Use of a sequential chimeric perforator flap for one-stage reconstruction of complex soft tissue defects of the extremities. Microsurgery.

[18] Koshima I, Inagawa K, Urushibara K, Moriguchi T (1998). Paraumbilical perforator flap without deep inferior epigastric vessels. Plast Reconstr Surg.

[19] Zhao D (2020). The development of microsurgery in China: retrospective and prospective views. Ann Plast Surg.

[20] Hong JP, Pak CJ, Suh HP (2021). Supermicrosurgery in lower extremity reconstruction. Clin Plast Surg.

[21] Hong JP (2009). The use of supermicrosurgery in lower extremity reconstruction: the next step in evolution. Plast Reconstr Surg.

[22] Homsy C, Theunissen T, Sadeghi A (2022). The thoracodorsal artery perforator flap: a powerful tool in breast reconstruction. Plast Reconstr Surg.

[23] Elgohary H, Nawar AM, Zidan A (2018). Outcome of pedicled thoracodorsal artery perforator flap in the surgical treatment of stage II and III hidradenitis suppurativa of axilla. Ann Plast Surg.

[24] Kulahci Y, Sever C, Uygur F (2011). Pre-expanded pedicled thoracodorsal artery perforator flap for postburn axillary contracture reconstruction. Microsurgery.

[25] Thomas BP, Geddes CR, Tang M, Williams J, Morris SF (2005). The vascular basis of the thoracodorsal artery perforator flap. Plast Reconstr Surg.

[26] Lee KT, Kim A, Mun GH (2016). Comprehensive analysis of donor-site morbidity following free thoracodorsal artery perforator flap harvest. Plast Reconstr Surg.

[27] Lin CT, Yang KC, Hsu KC (2009). Sensate thoracodorsal artery perforator flap: a focus on its preoperative design and harvesting technique. Plast Reconstr Surg.

[28] Kim SW, Youn DG, Kim JT, Kim YH (2018). A thoracodorsal artery perforator chimeric free flap for prevention of microvascular pedicle compression in lower extremity reconstruction. Microsurgery.

[29] Qing L, Wu P, Yu F, Zhou Z, Tang J (2019). Sequential chimeric deep circumflex iliac artery perforator flap and flow-through anterolateral thigh perforator flap for one-stage reconstruction of complex tissue defects. J Plast Reconstr Aesthet Surg.

[30] Yu J, Luo Z, Wu P, Tang J (2021). Novel design of the chimeric deep inferior epigastric artery perforator flap that provides for three-dimensional reconstruction of composite tissue defects of the heel in children. Orthop Surg.

[31] Pang X, Qing L, Wu P, Tang JY (2021). Catheter-based computer tomography angiography in double-skin paddle anterolateral thigh perforator. Ann Plast Surg.

[32] He J, Qing L, Wu P (2021). Individualized design of double skin paddle anterolateral thigh perforator flaps to repair complex soft tissue defects of the extremities: an anatomical study and retrospective cohort study. J Plast Reconstr Aesthet Surg.

[33] He J, Qing L, Wu P (2021). Large wounds reconstruction of the lower extremity with combined latissimus dorsi musculocutaneous flap and flow-through anterolateral thigh perforator flap transfer. Microsurgery.

[34] Qing L, Wu P, Liang J (2015). Use of flow-through anterolateral thigh perforator flaps in reconstruction of complex extremity defects. J Reconstr Microsurg.

[35] Qing LM, Tang JY (2019). Use of intraflap and extraflap microvascular anastomoses in combination for facilitating bipedicled DIEP/SIEA free flap for reconstruction of circumference soft tissue defect of extremity. Microsurgery.

[36] Xi S, Cheng S, Lou J (2019). A preliminary study of the effects of venous drainage position on arterial blood supply and venous return within the conjoined flap. Plast Reconstr Surg.

[37] Zhou ZB, Pan D, Wu PF (2018). Utilization of microdissected thin perforator flap technique in the treatment of bulky and deformed skin flaps. Ann Plast Surg.

[38] Guerra AB, Metzinger SE, Lund KM (2004). The thoracodorsal artery perforator flap: clinical experience and anatomic study with emphasis on harvest techniques. Plast Reconstr Surg.

[39] Kim KN, Hong JP, Park CR, Yoon CS (2016). Modification of the elevation plane and defatting technique to create a thin thoracodorsal artery perforator flap. J Reconstr Microsurg.

